# Pleiotropic Effects of Sitagliptin in the Treatment of Type 2 Diabetes Mellitus Patients

**DOI:** 10.4021/jocmr1061w

**Published:** 2012-09-12

**Authors:** Akira Kubota, Hajime Maeda, Akira Kanamori, Kiyokazu Matoba, Yasuyuki Jin, Fuyuki Minagawa, Mitsuo Obana, Koutarou Iemitsu, Shogo Ito, Hikaru Amemiya, Mizuki Kaneshiro, Masahiko Takai, Hideaki Kaneshige, Kazuhiko Hoshino, Masashi Ishikawa, Nobuaki Minami, Tetsuo Takuma, Nobuo Sasai, Sachio Aoyagi, Takehiro Kawata, Atsuko Mokubo, Hiroshi Takeda, Shin Honda, Hideo Machimura, Tetsuya Motomiya, Manabu Waseda, Yoshikazu Naka, Yasushi Tanaka, Yasuo Terauchi, Ikuro Matsuba

**Affiliations:** a The Study Group of the Diabetes Committee, Kanagawa Physicians Association, 3F Kanagawa-ken Sogo Iryo Kaikan, 3-1 Fujimi-cho, Naka-ward, Yokohama-city, Kanagawa 231-0037, Japan; b Department of Internal Medicine, Division of Metabolism and Endocrinology, St. Marianna University School of Medicine, 2-16-1 Sugao, Miyamae, Kawasaki, Kanagawa 216-8511, Japan; c Department of Endocrinology and Diabetes, Yokohama City University Medical Center, 4-57 Urafune-cho, Minami-ward, Yokohama-city, Kanagawa 232-0024, Japan

**Keywords:** Diabetes mellitus, GLP-1, Lipids, Sitagliptin, Pleiotropic effects

## Abstract

**Background:**

Sitagliptin is a DPP-4 inhibitor that became available for use in Japan three years ago. This study was conducted to identify the pleiotropic effects of sitagliptin other than blood glucose lowering in Japanese type 2 diabetes mellitus patients.

**Methods:**

A retrospective, observational study of 940 type 2 diabetes mellitus patients was conducted. The primary outcome measures were HbA1c, blood pressure, and lipid profiles measured at 0, 4, and 12 weeks of sitagliptin therapy.

**Results:**

After 12 weeks of sitagliptin treatment, compared with baseline, HbA1c decreased 0.64% ± 0.86%; systolic blood pressure (SBP) and diastolic blood pressure (DBP) decreased significantly; and serum creatinine (Cr) and uric acid (UA) levels were mildly but significantly elevated. A correlation analysis of the changes in systolic blood pressure, diastolic blood pressure, creatinine, and uric acid (ΔSBP, ΔDBP, ΔCr, ΔUA) from baseline to 12 weeks showed significant negative correlations between ΔSBP and ΔCr, ΔSBP and ΔUA, and ΔDBP and ΔCr. Total cholesterol and postprandial triglycerides were significantly decreased at both 4 and 12 weeks. Alkaline phosphatase (ALP) decreased significantly, and there was a significant positive correlation between changes in ALP and HbA1c.

**Conclusions:**

Sitagliptin seems to be effective not only in lowering blood glucose but also in lowering blood pressure, lipid, and ALP levels. Sitagliptin appears to contribute to a Na-diuretic action due to GLP-1.

## Introduction

Sitagliptin is a DPP-4 inhibitor that became available for use in Japan three years ago. It has been shown to be effective in Japanese diabetes mellitus patients [1, 2], who are characterized by a genetically low insulin secretory capacity [3, 4], and it is currently used in many such patients.

DPP-4 inhibitors administered from the early stage of diabetes help to protect pancreatic β cells and normalize glucagon secretion control, combining reliable hypoglycemic action and safety, inhibiting the progression of diabetes and maintaining glycemic control, along with the potential for cardiovascular protection and anti-arteriosclerotic action [5-8]. Incretin hormones are known to have diverse physiological actions, including acting on pancreatic islets of Langerhans [9, 10]. GLP-1 and GIP are secreted from L and K cells, respectively, in the small intestine when entry of nutrients to the gastrointestinal tract is detected. GLP-1 and GIP receptors are located not only on pancreatic β cells but are also distributed widely in the body, and GLP-1 and GIP have been found to have diverse physiological actions [9-12]. Consequently, when incretin hormones are activated by DPP-4 inhibitors in the treatment of diabetes, not only do they act to lower blood glucose, they may also bring about various other changes in the body. To demonstrate the pleiotropic effects of DPP-4 inhibitors in the body, large-scale collection of patient data and analysis are necessary. Our group, the Diabetes Committee of the Kanagawa Physicians Association, has previously published findings on the efficacy and safety of sitagliptin in large numbers of patients in actual clinical practice [13, 14].

The present study was a retrospective cohort study of type 2 diabetes mellitus patients who were given sitagliptin continuously for 12 weeks in actual clinical practice, to determine not only the hypoglycemic effect of sitagliptin but also its other pleiotropic effects.

## Materials and Methods

### Subjects

The survey subjects were type 2 diabetes mellitus patients receiving outpatient treatment at 28 hospitals or clinics specializing in diabetes belonging to the Diabetes Committee of the Kanagawa Physicians Association from December 2009 to August 2010.

Oral informed consent for participation in this study was obtained from type 2 diabetes mellitus patients who were using diet and exercise therapy and from patients with insufficient glycemic control despite the use of hypoglycemic agents. Sitagliptin monotherapy or sitagliptin in combination with other drugs was then given, starting at a dose of 25 mg or 50 mg. If good glycemic control was not obtained after this, the sitagliptin dose could be increased to 100 mg. To evaluate the effects of sitagliptin on blood pressure and lipids, the use of hypoglycemic agents and dyslipidemia medications was examined, excluding patients whose antihypertensive and lipid medications had been changed during the observation period. A total of 940 patients were registered. This study was an observational study that aimed to evaluate the efficacy of sitagliptin, and approval was obtained from the Ethics Review Board of the Kanagawa Physicians Association.

### Outcome measures

The patients’ baseline characteristics were as follows: 538 men, 402 women; mean age 63.3 ± 11.5 years; duration of diabetes 12.2 ± 8.0 years; and body mass index (BMI) 24.5 ± 4.3 kg/m^2^. The primary outcome measures, HbA1c, blood pressure, and lipid profiles, were measured at the start and after 4 and 12 weeks of sitagliptin therapy. HbA1c, expressed in National Glycohemoglobin Standardization Program (NGSP) units-equivalent value [15], was measured by high-performance liquid chromatography.

The 940 patients included 178 patients who received sitagliptin monotherapy and 762 patients who received combination therapy that included other oral medications. Co-administered drugs in the combination therapy group were analyzed, and the numbers of patients taking sulfonylureas, biguanides, pioglitazone, αGI, and glinides were 620, 420, 210, 84 and 13, respectively. HbA1c was analyzed at baseline and after 4 and 12 weeks, and body weight was analyzed at baseline and after 12 weeks.

### Statistical analysis

All analyses were performed using SPSS version 19 for Windows. For parameters during sitagliptin treatment, data were analyzed by one-way ANOVA. The correlations between variables were assessed by Pearson’s correlation coefficient. In correlation analysis, Δ was used as the difference in each variable between the start of sitagliptin and after 12 weeks. Data are presented as means ± SD. A P value < 0.05 was considered significant.

## Results

Blood pressure was analyzed after the start of sitagliptin in 940 patients, including 619 in whom antihypertensive agents were not administered from before the start of sitagliptin until 12 weeks afterward, and 321 in whom antihypertensive agents were administered but the agent was not changed during the same observation period. With the administration of sitagliptin, HbA1c decreased significantly from 8.02% ± 1.12% at baseline to 7.71 ± 0.98% at 4 weeks (P < 0.01) and 7.38% ± 0.89% at 12 weeks (P < 0.01). Systolic blood pressure (SBP) decreased significantly from 128.8 ± 14.3 mmHg at baseline to 127.6 ± 13.7 mmHg at 4 weeks (P < 0.05) and 127.4 ± 13.4 mmHg at 12 weeks (P < 0.01). During the 12 weeks, significant changes were seen in diastolic blood pressure (DBP), which went from 74.7 ± 9.8 mmHg at baseline to 74.0 ± 9.6 mmHg at 4 weeks and 74.0 ± 9.3 mmHg at 12 weeks (P < 0.01).

Of the lipids, total cholesterol (TCHOL) decreased significantly from 198.2 ± 34.3 mg/dL at baseline to 192.6 ± 34.3 mg/dL at 4 weeks (P < 0.01) and 191.7 ± 32.5 mg/dL at 12 weeks (P < 0.01). HDL did not change significantly after sitagliptin administration. Triglycerides were analyzed with patients divided into a postprandial measurement group and a fasting measurement group. No significant change was seen in fasting triglycerides (fTGs), but postprandial triglycerides (ppTGs) decreased significantly from 157.4 ± 99.4 mg/dL before the start of sitagliptin administration to 144.9 ± 87.2 mg/dL at 4 weeks (P < 0.05) and 146.3 ± 86.8 mg/dL at 12 weeks (P < 0.05).

Although the changes in serum creatinine (Cr) and uric acid (UA) after sitagliptin administration were within the normal range, they rose significantly from 0.75 ± 0.22 mg/dL at baseline to 0.78 ± 0.24 mg/dL at 12 weeks and 5.07 ± 1.21 mg/dL at baseline to 5.40 ± 1.45 mg/dL at 12 weeks, respectively. Correlations among changes in systolic blood pressure, diastolic blood pressure, creatinine, and uric acid (ΔSBP, ΔDBP, ΔCr, ΔUA) between baseline and 12 weeks were analyzed (Table 1). The results showed a positive correlation between ΔCr and ΔUA, and significant negative correlations between ΔSBP and ΔCr, ΔSBP and ΔUA, and between ΔDBP and ΔCr. Correlation analysis revealed a significant positive correlation between ΔALP and ΔHbA1c (Table 1).

**Table 1:** Univariate Correlation Between Changes of Parameters During 12 Weeks’ Sitagliptin Treatment

		ΔCr	ΔUA
ΔSBP	r	-0.236	-0.134
	P	< 0.01	< 0.05
ΔDBP	r	-0.161	-0.079
	P	< 0.01	NS
ΔCr	r		0.342
	P		< 0.01

			

		**ΔHbA1c**	

ΔALP	r	0.383	
	P	< 0.01	

Correlations among blood pressure, creatinine, and uric acid levels; correlation between changes in ALP and HbA1c.

Serum alkaline phosphatase (ALP) was also found to decrease significantly after sitagliptin administration, from 255.3 ± 93.0 IU/L at baseline to 240.3 ± 86.1 IU/L at 4 weeks (P < 0.01) and 231.8 ± 78.2 IU/L at 12 weeks (P < 0.01) (Table 2). No significant changes were seen in GOT, GPT, γGTP or LDH.

**Table 2:** HbA1c, Blood Pressure, Lipid Profile, and Laboratory Data Over Time During Sitagliptin Treatment

	0 weeks	4 weeks	12 weeks
HbA1c (%)	8.02 ± 1.12	7.71 ± 0.98 **	7.38 ± 0.89 **,††
SBP (mmHg)	128.8 ± 14.3	127.6 ± 13.7*	127.4 ± 13.4**
DBP (mmHg)	74.7 ± 9.8	74.0 ± 9.6	74.0 ± 9.3*
TCHOL	198.2 ± 34.3	192.6 ± 34.3**	191.7 ± 32.5**
HDL (mg/dL)	57.1 ± 15.2	56.4 ± 14.9	56.2 ± 14.8
ppTGs (mg/dL)	157.4 ± 99.4	144.9 ± 87.2*	146.3 ± 86.8*
fTGs (mg/dL)	132.4 ± 141.2	125.0 ± 107.4	136.9 ± 125.2
Cr (mg/dL)	0.75 ± 0.22	0.77 ± 0.23**	0.78 ± 0.24**
UA (mg/dL)	5.07 ± 1.21	5.28 ± 1.32**	5.40 ± 1.45**
ALP (IU/L)	255.3 ± 93.0	240.3 ± 86.1**	231.8 ± 78.2**
GOT (IU/L)	25.3 ± 15.9	25.5 ± 18.6	25.3 ± 18.0
GPT (IU/L)	27.7 ± 21.2	26.3 ± 21.7	26.9 ± 22.8
γGTP (IU/L)	45.7 ± 50.4	44.1 ± 55.3	44.8 ± 54.9
LDH (IU/L)	244.6 ± 63.2	249 ± 60.2	246 ± 52.6

ANOVA: vs. baseline ** P < 0.01 * P < 0.05 vs. 4 weeks †† P < 0.01.

## Discussion

The results of a survey of 940 Japanese type 2 diabetes mellitus patients treated with sitagliptin showed that not only blood glucose but also blood pressure decreased significantly with sitagliptin therapy. Compared with the start of treatment, significant decreases of 1.4 mmHg in systolic pressure and 0.78 mmHg in diastolic pressure were seen at 12 weeks. There are still few reports on the effects of sitagliptin on blood pressure. Mistry [16] and Ogawa et al [17] reported that blood pressure decreased with sitagliptin administration, which corresponds with the present results. These reports did not identify the mechanism for the decrease in blood pressure with sitagliptin. Interestingly, in the present study, sitagliptin increased serum creatinine and uric acid levels significantly, although the increase was slight, and the changes in serum creatinine and uric acid (ΔCr and ΔUA) had a significant positive correlation. GLP-1 has been shown to have a Na-diuretic action [18], and it is well known that serum creatinine and uric acid levels increase slightly when a Na-diuretic action occurs. This suggests the possibility that a Na-diuretic action due to GLP-1 occurs with sitagliptin therapy. It was also shown that the change in blood pressure after administration of sitagliptin was significantly negatively correlated with the changes in Cr and uric acid. Together, these results suggest that decreases in blood pressure in patients who receive sitagliptin occur due to a Na-diuretic action.

It was also shown that both TCHOL and ppTGs decreased significantly with sitagliptin treatment. In a meta-analysis of the effects of DPP-4 inhibitors on lipids, Monami et al showed that TCHOL decreased with DPP-4 inhibitor treatment [19]. A decrease in postprandial triglycerides with sitagliptin therapy was also reported by Tremblay et al [20] Multiple action mechanisms are assumed for the tryglyceride-lowering effect of sitagliptin, including inhibited TG absorption from the intestines, inhibited VLDL release from the liver, and decreased blood glucose levels and accompanying improvements in metabolic status due to GLP-1. In actual clinical practice, it is difficult to evaluate postprandial changes in triglycerides, but it is thought that significant movements could be detected in this study through observations of a large number of patients. The effect of DPP-4 inhibitors on cardiovascular events has not yet been determined, but in studies including a meta-analysis, DPP-4 inhibitors were shown to have an inhibiting effect on cardiovascular events [21, 22]. In the present study of actual clinical practice, sitagliptin was shown to lower blood pressure and lipid levels, which is an important finding linked to the pleiotropic effects of sitagliptin in inhibiting cardiovascular events.

It was also interesting that, in this observational study, ALP was significantly decreased after sitagliptin therapy. In fact, there is a past report of decreased ALP with sitagliptin therapy [23]. The significant correlation of the decrease in ALP with the decrease in HbA1c in the present study suggests the possibility that the effect on ALP is a change accompanying the action of sitagliptin, in particular activation of incretin. ALP is known to be present in the liver, biliary tract, bones, small intestine, and elsewhere, but in this study, ALP isozymes were not measured, so that one cannot judge the type of tissue in which the changes occurred. So far it has been shown that sitagliptin improves fatty liver [24, 25], that GIP contributes to bone metabolism [26, 27], and, from a meta-analysis, that the risk of bone fracture is significantly reduced in patients treated with DPP-4 inhibitors [28]. Further analysis of the liver and bones in particular is thought to be necessary to determine the physiological significance of the changes in ALP.

Since the present study was an observational study without controls, there are limits to the interpretation of results. However, unlike prospective studies, it is thought that the findings reflect the effects of sitagliptin in actual clinical practice. The large number of subjects in the analysis is another feature of this study, and the results were also statistically significant. The present retrospective cohort study showed that sitagliptin in actual clinical practice has pleiotropic effects other than lowering blood glucose.
